# Shared auditors, information asymmetry degree, and mergers and acquisitions value creation

**DOI:** 10.3389/fpsyg.2022.921581

**Published:** 2022-08-26

**Authors:** Chunhui You

**Affiliations:** School of Economics and Management, Jiaying University, Meizhou, China

**Keywords:** shared auditors, information asymmetry, related M&A, industry attributes, M&A value creation

## Abstract

The social network is an important factor that affects the value creation of mergers and acquisitions (M&A). The M&A events of China’s Listed Companies in 2011–2018 were used as research samples, and this study used the ordinary least square method to test the value creation effect of shared auditors. First, it analyzed the impact of shared auditors on the current, short-term, and long-term M&A value creation. Second, it analyzed the moderating effect of information asymmetry degree. The research results show that shared auditors can increase the enterprise value of both sides of M&A. In addition, related M&A and industry attributes can moderate the relationship between shared auditors and M&A value creation. When the M&A are unrelated and in different industries, shared auditors play a more significant role in the value creation of M&A that have more asymmetric information.

## Introduction

Mergers and acquisitions (M&A) are not only important ways of allocating market resources but are also an important way for enterprises to rapidly grow. Theoretically, M&A can create value for shareholders through synergy effect, scale effect, and market advantages, but the reality is that a large number of enterprises weaken their performance and damage their wealth owing to M&A. One of the main reasons is that there is information asymmetry between both sides of the M&A (Cai and Sevilir, 2012; Chen et al., 2013). The acquirer and target enterprises are affected by the differences in interest demands, industries, and regions, which can provide both sides of the acquirer and target enterprises with the motivation and possibility to hide internal core information and even disseminate false information. Therefore, obtaining the real financial status, management level, R&D ability, and other information of the other party has become the key to success of M&A and the creation of M&A value.

It is common for both sides of M&A to employ the same accounting firm [i.e., a shared auditor (SA)] (Zheng and Zhu, 2021). The existence of SAs enables accounting firms to simultaneously master the important information of both sides. Can accounting firms alleviate the degree of information asymmetry between both sides and improve the performance of M&A during the process of auditing a business? Previous studies have shown that SAs can reduce the uncertainty of M&A, increase the information flow of both sides, and increase the comparability of financial statements (Cai et al., 2016; Dhaliwal et al., 2016; Chircop et al., 2018; Bedford et al., 2022). However, these studies are inconsistent with the conclusion of SAs on the performance of both sides of M&A. For example, Cai et al. (2016) found that SAs reduced the uncertainty during the process of M&A, and the quality of M&A was higher. However, Dhaliwal et al. (2016) and Bedford et al. (2022) found that the benefits of SAs in reducing the uncertainty of M&A only benefitted the acquirer. Simultaneously, the existing literature utilizes mature capital markets in developed countries as the research object and ignores the research on weak form efficient capital markets.

The research of this study has made several important contributions. First, this study investigated how SAs affect the performance of M&A in weak form efficient capital markets, which enriches the scope of application of the conclusions on SAs. Second, the existing studies use the cumulative abnormal return (CAR) of the capital market, Tobin Q, ROA, or principal component analysis, to measure the performance of M&A. From the perspective of M&A value creation, this study adopted economic value added (EVA) series indicators, which can help investors to more clearly observe the incremental value of M&A. Third, starting from the social network theory, this study investigated the information bridge role of SAs, integrated social networks with corporate finance, and enriched the literature in the field of social networks.

The rest of this study is organized as follows: This study first discusses the previous literature and the development of hypotheses and then describes the methods used in this study, including sample selection and study design. In the “Results” section, it discusses the empirical results of the impact of SAs on M&A value creation. Finally, the study is summarized.

## Literature review and hypothesis development

### Literature review

Whether M&A can increase enterprise value and how to increase enterprise value are important issues in M&A research. Currently, there are still disputes about whether M&A can increase enterprise value. There are basically four types of views that include increase theory, uncertainty theory, fluctuation theory, and decline theory. Although the research conclusions on the economic consequences of M&A are inconsistent, the existing studies have generally concluded that an important reason for the decline of enterprise value caused by M&A is the information asymmetry between the two sides of M&A (Chen et al., 2013; Li et al., 2019).

A social network is an important way for enterprises to survive and develop. It can be used as a bridge for communication and exchange of resources between both sides of the M&A, reduce the degree of information asymmetry (Huang and Li, 2019), and significantly affect the performance of M&A. Its forms of existence include the network formed by the upstream and downstream relationships of enterprises and those of the director, shareholder, alumni, and kinship networks (Liu et al., 2015; Jiang and Zhang, 2019). In fact, the relationship network of accounting firms is also an important form of enterprise social network, which is widespread and will affect the behavior and economic consequences of enterprises. SAs have been found to play a role in the following aspects:

(1) Enterprise supply chain. Cai et al. (2019) found that SAs will affect the cost stickiness of suppliers. When the managers of suppliers are optimistic about future, SAs in the supply chain reduce the cost stickiness of suppliers, which vice versa, will increase the cost stickiness of suppliers. Hu et al. (2022) found that SAs enhance the trust between suppliers and customers and improve the investment of suppliers in specific relationships.
(2) The cost of bank loans and default behavior. SAs help banks verify the financial information of borrowers and reduce the debt cost of loan enterprises and the possibility of future defaults (Aguir et al., 2022).
(3) Analyst information forecast. When security companies and enterprises employ the same accounting firm, these firms become one of the sources of forecast information for analysts under security companies (Liu and Xie, 2017).
(4) Mergers and acquisitions. Nearly one-quarter of M&A have SAs. SAs can significantly reduce the M&A premium with a higher return on investment and a higher transaction completion rate (Dhaliwal et al., 2016). The role of SAs is more significant when there is more uncertainty in the M&A (Cai et al., 2016; Chircop et al., 2018; Bedford et al., 2022).

A review of the relevant literature on SAs shows that they are a common phenomenon and have received continuous attention from researchers. In the related research of corporate M&A, the existing literature has utilized CAR to measure the M&A performance of SAs from the perspective of market reflection. In the mature capital market, valuable information can be timely and fully reflected in the stock price trend. However, in a weak form efficient market, the sufficiency, timeliness, and effectiveness of information are insufficient, and the legal and investor protection mechanism is imperfect. If CAR is used to measure the M&A performance of SAs, it could lead to a deviation in the conclusions of the research. EVA represents the real economic benefits brought by assets rather than the market’s view of value growth, and it can be used to measure the real value creation of M&A in weak form efficient markets. Although China’s capital market is developing rapidly, it is still a weak form efficient market. Therefore, this study attempts to examine the effect of SAs in M&A of the Chinese Listed Companies from a financial perspective using the EVA series of indicators and consider the impact of the degree of information asymmetry to enrich the research on SAs and M&A performance.

### Hypothesis development

Enterprises are heterogeneous aggregates that are composed of different resources, and their valuable, scarce, irreplaceable, and difficult-to-imitate resources are the basis for enterprises to maintain a long-term competitive advantage (Barney, 1991; Chalenon et al., 2017; Yu and Li, 2019; Chiu et al., 2022). The heterogeneous resources with competitive advantages owned by enterprises could become the core rigid factor to resist change because they have difficulty adapting to the changes of time and environment. Enterprises need to constantly search for and expand new resources to maintain their competitive advantage (Helfat and Peteraf, 2003). Whether the resources obtained by enterprises can bring value and how much value they can bring are uncertain before the completion of a business merger, and it can be finally determined only after the merger has been completed (Makadok, 2001). Therefore, fully and accurately obtaining and identifying the priority and asymmetric information of the future value of available resources is the key to the proper selection of the M&A targets (Palepu, 1986; Chen et al., 2013).

Social network theory considers the whole society to be a large system that is composed of interlaced or parallel networks. The strength of social relations can be divided into strong and weak, and weak relations exist between groups and organizations. Compared with strong relationships, weak relationships can cross social boundaries and become a bridge for groups and organizations to obtain information and resources, that is, weak relationships play the role of an information bridge, which can help economic actors quickly find trading opportunities in the market and transfer this information to the market with near zero risk (Granovetter, 1973; Burt, 1997). The function of social networks to transmit information is more efficient and reliable than the traditional information channels, such as government, market research, guild organization, and commercial espionage, and has become the most important and valuable information source for enterprises (Sharma and Blomstermo, 2003; Wang et al., 2021). For example, Chaudhry et al. (2022) found that financial consulting companies play the role of extracting and disseminating information, and Barros et al. (2021) also found that board interlocking reduces the degree of information asymmetry in enterprise M&A. While they provide audit services for the acquirer and target enterprises, SAs also build a bridge between them, thus, building a social relationship network.

SAs could become a high-quality source of M&A information for both parties. There are several reasons for this. First, accounting firms have a guaranteed reputation. Accounting firms audit businesses as an independent third party, which is highly professional, objective, and fair, and plays a key role as “gatekeeper” in the capital market. In recent years, with the marketization of Chinese enterprise M&A businesses, accounting firms have become one of the intermediaries of M&A, and their role and influence in M&A have been recognized by the market.^1^ Therefore, the information provided by accounting firms is often highly reliable. Second, auditing is a high-quality assurance business whose purpose is to find and report misstatements and illegal acts of the audited entity. Owing to the need to reasonably ensure that there are no material misstatements in the financial statements of listed companies as a whole, accounting firms usually take a long time to implement an audit, and their materials and information are more comprehensive. This enables them to more thoroughly understand the financial status and operating capacity of the entity audited. M&A events have a significant impact on the entity audited. The operating risk of listed companies with M&A is often higher than that of listed companies without M&A (Pan and Chen, 2005). To reduce the risk of an audit, CPAs should deeply communicate with management and governance on M&A transactions.^2^ When the acquirer and target enterprises share the same auditor, the accounting firm that performed the audit business can obtain unpublished detailed information and cutting-edge information related to the M&A of both parties (Yang et al., 2015). When communicating with the auditee on M&A matters, the CPAs may inadvertently disclose some of the other party’s key M&A information. Third, China is a typical relational society, and social relations constitute the link of interest relations between people (Liu and Xie, 2017). Usually, enterprises will hire the same accounting firm for a long time, so there is a close relationship between the two. Simultaneously, listed companies are usually high-quality customers, and accounting firms will consciously maintain the relationship with the auditee to improve their likelihood of being re-employed. When the auditee enquires about the other party’s relevant information, the CPAs could also disclose some information under the pressure to maintain a good relationship (Dhaliwal et al., 2016). As the “matchmaker” between the two sides of M&A, CPAs can alleviate information asymmetry, help to understand the real ideas of the other party’s decision-makers before M&A, enhance trust and reduce differences in negotiation; accurately estimate the value of the target enterprise and the possibility of accepting the offer and avoiding excessive payment in M&A; and more effectively match resources and improve the success rate of integration after M&A. Combined with the analysis described earlier, I formally state my first hypothesis as follows:

Hypothesis 1: When the other conditions remain unchanged, SAs can improve the performance of both sides of the M&A.

According to hypothesis 1, SAs can alleviate the degree of information asymmetry and improve the synergy of M&A. If the degree of information asymmetry between the two sides of M&A is higher, the intermediary role of SAs in providing information should be more apparent. Therefore, the following analysis will test the mechanism of SAs from the perspectives of related M&A and industry attributes.

A considerable number of listed companies in China are restructured from state-owned enterprises and asset stripping leads to countless links between the listed and parent companies. Simultaneously, because related M&A can save transaction costs, they can quickly improve the operating performance of listed companies and manipulate the stock trading price in the secondary market (Pan and Chen, 2005). There are a large number of related M&A transactions in Chinese listed companies. Compared with related M&A, most of the unrelated M&A adopt market behavior, follow market laws and rules, and generally do not practice “tunnel behavior” among the major shareholders. However, there are many disadvantages in the implementation of unrelated M&A. First, there is a lack of trust mechanism between the two sides of unrelated M&A. Distrust can substantially reduce the authenticity and efficiency of transmitting information and increase the acquisition and transaction costs of mutual information between M&A parties. Second, acquirer enterprises lack a comprehensive understanding of the actual situation of the target enterprises, which makes it difficult to obtain and absorb M&A resources and improve output (Hussinger, 2012). Third, there may be cultural differences between the acquirer and target enterprises, resulting in obstacles for the integration of human resources and business after the M&A has taken place (Tong et al., 2018). All the disadvantages originate from the information asymmetry between the two sides of M&A. In the case of SAs, CPAs who implement the audit act as the information intermediary between unrelated M&A enterprises, which can alleviate the degree of information asymmetry and improve the M&A output. Based on this, I propose the second hypothesis as follows:

Hypothesis 2: Compared with related M&A, unrelated M&A enterprises that use SAs have a more significant effect on the M&A value creation.

The specialization and fine division of labor in modern society increase the span and heterogeneity between industries. The suppliers and customers of enterprises in the same industry are basically the same and have similar product markets, so there are more channels to obtain each other’s private information (Han et al., 2014). The homogeneity of resources enables the M&A parties to more effectively integrate various resources among enterprises and realize the three synergistic effects of the operation, finance, and collusion (Zhou and Li, 2008). In contrast, enterprises in different industries have substantial differences in their business scope, business strategy, organizational culture, management mode, and focus on innovation (Lu and Dang, 2014). In addition, the lack of a mechanism to communicate information makes it more difficult to obtain information on the other party’s real situation. This makes it easy for the phenomenon to appear that results in the differences in the profession that makes one feel incompatible. To reduce the cost of information acquisition and avoid integration failure, both sides of M&A prefer to obtain each other’s information through their own relationship network. The existence of an SA as an intermediary channel for information will be more important to alleviate the degree of information asymmetry between both sides of M&A. This leads to my third hypothesis:

Hypothesis 3: Compared with the same industry, enterprises in different industries that employ SAs have a more significant effect on M&A value creation.

## Data and study design

### Data

The data of this study originate from the CSMAR database of Guotai’an. Based on the sample of Chinese A-share listed companies that completed M&A transactions from 2011 to 2018, this study conducted the following screening: (1) excluded financial listed companies; (2) excluded the sample of M&A failures; (3) only retained equity acquisition transactions and excluded asset acquisition transactions, such as land and asset purchases; (4) when listed companies have completed M&A transactions many times in a year, only the first M&A completed by the company in that year was selected as the sample; (5) only samples in which both the acquirer and the target companies that are listed were retained; and (6) samples with missing data were excluded. The processing described above resulted in 316 valid samples.

### Study design

To test hypothesis 1, the results of Cai et al. (2016) and Chen and Xing (2018) were used to construct an ordinary least squares (OLS) regression analysis model as follows:


(1) $$\text{VC}=\beta_{0}+\beta_{1}\,\text{SA}+\gamma \,\text{Controls}+\text{Year}\,\mathrm{FE}+\mathrm{Industry}\,\mathrm{FE}+\epsilon$$

Where VC represents the M&A value creation, which is measured by EVA series indicators, including fixed effects (FE). There were several reasons why the EVA index was chosen to investigate the M&A value creation. First, China’s capital market is still a weak form efficient market, and the market research method could lead to errors in the results. Second, any investment can only be called value creation if the final income is greater than the cost. However, the traditional financial performance evaluation indicators, such as the return on assets (ROA) and the earnings per share (EPS), do not exclude the cost of invested capital and cannot accurately measure value creation. EVA is the difference between the net operating profit after tax and the total cost of capital. On the one hand, it considers the income and cost of the enterprise, which leads to results that are more real and reliable. Alternatively, it makes accounting adjustments to construction in progress, R&D expenses, deferred income tax, and goodwill, which can objectively and truly reflect the value creation ability of the enterprise to the fullest extent (Lu, 2012). To investigate the impact of SAs on M&A value creation in more detail, this study utilized the practices of Ge (2015) to observe this phenomenon from three perspectives: the EVA of both M&A parties in the year of the merger, the difference between the EVA of the merger year and the previous year (i.e., short-term M&A value creation), and the difference between the EVA of the next year and the previous year (i.e., long-term M&A value creation). Among them, MEVA (TEVA) is the EVA of the acquirer enterprise (target enterprise), which is calculated based on the natural logarithm of EVA in the year that the date of the acquirer enterprise (target enterprise) was first announced. ΔMEVA_(–1,0)_ [ΔTEVA_(–1,0)_] is the short-term M&A value creation of the acquirer enterprise (target enterprise), which is equal to the natural logarithm of EVA in the year of the first date of announcement minus the natural logarithm of EVA in the year before the announcement. ΔMEVA_(–1,1)_ [ΔTEVA_(–1,1)_] is the long-term M&A value creation of the acquirer enterprise (target enterprise), which is equal to the natural logarithm of EVA in the next year after the first announcement minus the natural logarithm of EVA in the year before the announcement.

Cai et al. (2016) and Dhaliwal et al. (2016) define an auditor as an SA when the same firm is employed by the acquirer and target enterprises. Since the audit business of the enterprise in that year is primarily conducted at the beginning of the next year, to more accurately reflect the impact of SAs on the value creation of M&A, this study established the parameter that when the last year and this year of M&A were the same accounting firm (i.e., the audit engagement period covered the actual completion date of the M&A event), it was determined to be an SA. As previously described (Dhaliwal et al., 2016; Chen and Xing, 2018), this study also includes the following control variables: whether to make cash payments (CP), when the M&A payment method is cash, it is 1; otherwise, it is 0; the total return on assets (ROTA), which is equal to the sum of total profits and financial expenses divided by total assets; equity multiplier (EM), which is equal to the total assets divided by total owner’s equity; total income (TI) is the natural logarithm of the total income; the shareholding ratio of the largest shareholder (LS), which is the shareholding ratio of the LS of the listed company; book to market ratio (BM) is equal to the total assets divided by the market value; and whether it is a state-owned holding company (SH), it is 1 when the listed company is state-owned, otherwise it is 0. To reduce the possible endogenous problems in the model, this study treated the enterprise characteristic variables with a lag period. This study also simultaneously controlled the industry and annual variables.

## Results

### Descriptive statistics

The descriptive statistical results are shown in Table 1. From the dependent variable indicators, on average, the EVA indicators of both M&A parties in the observed samples were positive as a whole, but there was a large standard deviation (*SD*), indicating that there were substantial differences between the different samples. Moreover, in the short-term M&A value creation and long-term M&A value creation indicators, some enterprises had negative values. However, the average value was greater than 0, indicating that the overall EVA of the observed samples had increased compared with that before the M&A. The average value of the SA index was 0.39, which indicated that 39% of the listed companies in the sample had SAs, which presents a good research foundation for this study. The average value of CP was 0.96, indicating that most M&A samples adopted the method of CP.

**TABLE 1:** Descriptive statistics.

(A) M&A variables						
VarName	Obs	Mean	*SD*	Min	Max	Median
MEVA	316	18.83	1.75	10.03	23.48	18.99
TEVA	316	18.79	1.74	10.04	23.17	18.95
ΔMEVA_(–1,0)_	257	0.03	1.10	–6.94	4.29	0.15
ΔTEVA_(–1,0)_	257	0.02	1.04	–6.94	3.58	0.17
ΔMEVA_(–1,1)_	221	0.10	1.16	–4.16	5.37	0.15
ΔTEVA_(–1,1)_	221	0.14	1.02	–3.62	5.10	0.20
SA	316	0.39	0.49	0.00	1.00	0.00
CP	316	0.96	0.19	0.00	1.00	1.00

**(B) Characteristics of acquirer enterprise**						
**VarName**	**Obs**	**Mean**	***SD***	**Min**	**Max**	**Median**

ROTA	316	0.07	0.40	–0.92	11.01	0.05
EM	316	1.04	0.03	1.00	1.24	1.03
TI	316	21.64	1.53	15.87	26.00	21.57
LS	316	0.32	0.15	0.04	0.84	0.29
BM	316	0.59	0.26	0.01	1.20	0.60
SH	316	0.52	0.50	0.00	1.00	1.00

**(C) Characteristics of target enterprise**						
**VarName**	**Obs**	**Mean**	***SD***	**Min**	**Max**	**Median**

ROTA	316	0.09	0.06	–0.18	0.33	0.08
EM	316	1.03	0.02	1.00	1.09	1.03
TI	316	21.87	1.34	16.80	25.66	21.78
LS	316	0.33	0.15	0.07	0.81	0.29
BM	316	0.55	0.25	0.06	1.13	0.54
SH	316	0.47	0.50	0.00	1.00	0.00

This study determined the Spearman correlation coefficient between the variables. The statistical results of the correlation coefficient show that SA significantly positively correlated with the EVA and short-term M&A value creation of both parties (*P* < 0.01). The SA positively correlated with the long-term M&A value creation, but it was not significant. The correlation coefficient between each variable was less than 0.5, and the maximum value of variance inflation factor (VIF) in the statistical results was 1.94, indicating that the multicollinearity problem was not serious.

### Empirical results

Table 2 reports the regression results of SAs on the M&A value creation of the acquirer and target enterprises. In columns (1) and (4), the coefficients (T values) of SA were 0.051 (2.40) and 0.047 (1.67), respectively, and they significantly correlated at the 5% and 10% levels, respectively. Since EVA represents the value created for shareholders by enterprises, the results described above show that the SAs increased the value created for shareholders by both enterprises in the current year. In columns (2) and (5), the coefficients (T values) of SA were 0.058 (2.93) and 0.088 (3.73), respectively, (*P* < 0.01). ΔMEVA_(–1,0)_ and ΔTEVA_(–1,0)_ represent the difference between the EVA of the current year and that of the previous year. Thus, these statistical results indicate that SAs bring incremental value to both parties in the short term. In columns (3) and (6), although the coefficient of SA was positive, it was not significant, indicating that the long-term value creation effect of SAs is not tenable. This conclusion is consistent with those of Feng and Wu (2001) and Lu (2012) that the performance of M&A increases first and then decreases. The reason could be that in reality, enterprise managers often pay attention to M&A but despise management and integration, resulting in the short-term value creation effect of M&A. Hypothesis 1 has been partially verified.

**TABLE 2:** Shared auditors and M&A value creation.

Variables	Acquirer enterprise			Target enterprise		
	(1) MEVA	(2) ΔMEVA_(–1,0)_	(3) ΔMEVA_(–1,1)_	(4) TEVA	(5) ΔTEVA_(–1,0)_	(6) ΔTEVA_(–1,1)_
SA	0.051** (2.40)	0.058*** (2.93)	0.006 (0.32)	0.047* (1.67)	0.088*** (3.73)	0.022 (0.85)
CP	0.109*** (2.76)	0.045 (1.23)	0.042 (1.03)	0.082** (2.31)	0.042 (1.24)	0.042 (1.04)
ROTA	0.798*** (4.68)	–0.529*** (-3.36)	–0.733*** (-4.61)	0.756*** (4.78)	–0.506*** (-3.28)	–0.720*** (-4.44)
EM	–0.442 (-0.84)	–0.368 (-0.76)	–0.040 (-0.08)	0.002 (0.10)	–0.203 (-0.44)	0.010 (0.02)
TI	0.098*** (12.50)	0.013* (1.78)	0.005 (0.68)	0.078*** (10.00)	0.010 (1.38)	0.002 (0.31)
LS	0.100** (2.14)	0.078* (1.81)	0.024 (0.57)	0.102** (2.05)	0.086** (2.01)	0.053 (1.20)
BM	–0.024 (-0.55)	–0.082** (-2.10)	–0.081* (-2.05)	–0.033 (-0.76)	–0.067* (-1.76)	–0.089** (-2.22)
SH	–0.013 (-0.85)	–0.005 (-0.38)	–0.012 (-0.81)	–0.025 (1.62)	0.003 (0.20)	–0.004 (-0.27)
Constant	12.190 (0.91)	23.460 (1.90)	–2.107 (-0.17)	–0.399 (-0.03)	31.010** (2.38)	5.077 (0.36)
Year fixed effect	Yes	Yes	Yes	Yes	Yes	Yes
Industry fixed effect	Yes	Yes	Yes	Yes	Yes	Yes
*N*	316	257	221	316	257	221
*R* ^2^	0.569	0.094	0.144	0.480	0.125	0.142

t-statistics are reported in parentheses and are based on robust standard errors clustered at the firm level. *P < 0.10. **P < 0.05. ***P < 0.01.

Among the control variables, the coefficient of CP significantly positively correlated with the current EVA of both M&A parties, which could be because cash is an asset that can be readily liquidated but is not likely to be profitable. Using enterprise idle cash for M&A investment can reduce the capital occupation cost and improve efficiency. Alternatively, raising M&A funds through new liabilities can reduce the weighted average cost of capital and create value for shareholders. Overall, the greater the rate of return on total assets (ROTA), total income (TI), and the shareholding ratio of the largest shareholder (LS) results in a more apparent effect of M&A value creation in the current period. However, this effect was not significant for the value creation of long-term M&A. The book-to-market ratio (BM) negatively correlated with the short-term and long-term M&A value creation.

To test hypotheses 2 and 3 based on model (1), this study multiplied the variables of related M&A (RM) and industry attribute (IA) by SA and analyzed the moderating effect of related M&A and industry attributes according to the coefficient and significance of the interaction term, which established the OLS model (2). If the M&A transaction did not occur between the listed company and its controlling shareholder group, RM is assigned 1; otherwise, it is assigned 0. If the M&A transaction did not occur in the same industry, IA is assigned 1; otherwise, it is assigned 0.


(2) $$\text{VC}=\beta_{0}+\beta_{1}\,\text{SA}+\beta_{2}\,\text{RM}\,\left(\text{IA}\right)+\beta_{3}\,\text{SA}*\text{RM}\,\left(\text{IA}\right)+\gamma \,\text{Controls}+\text{Year}\,\mathrm{FE}+\mathrm{Industry}\,\mathrm{FE}+\epsilon$$

Table 3 reports the moderating effect of related M&A on the relationship between SA and M&A value creation. The coefficient of RM was negative, but it was only significant with columns (1), (3), and (6), which could be owing to the serious information asymmetry between unrelated M&A parties, which affects the performance of M&A. SA *RM in columns (1) and (4) significantly positively correlated with the current EVA of both M&A parties (*P* < 0.01), and SA *RM in columns (2) and (5) significantly positively correlated with the short-term M&A value creation of both M&A parties (*P* < 0.05), indicating that in the case of unrelated M&A, the effect of shared auditors on the current EVA and short-term M&A value creation of both M&A parties was more significant. Simultaneously, we observed that the coefficients of SA *RM in columns (3) and (6) were also significantly positively correlated (*P* < 0.05). These statistical results show that the SAs alleviated the degree of information asymmetry between unrelated M&A enterprises and had a more significant value creation effect than related M&A. Thus, hypothesis 2 was verified.

**TABLE 3:** Moderating effect of related M&A.

Variables	Acquirer enterprise			Target enterprise		
	MEVA (1)	ΔMEVA_(–1,0)_ (2)	ΔMEVA_(–1,1)_ (3)	TEVA (4)	ΔTEVA_(–1,0)_ (5)	ΔTEVA_(–1,1)_ (6)
SA	0.044** (2.05)	0.053** (2.05)	0.002 (0.10)	0.041 (1.48)	0.085*** (3.33)	0.019 (0.79)
RM	–0.077* (-1.91)	–0.065 (-1.54)	–0.108*** (-4.01)	–0.049 (-1.36)	–0.062 (-1.56)	–0.102*** (-3.63)
SA*RM	0.204*** (3.47)	0.160** (2.01)	0.135** (2.24)	0.238*** (4.14)	0.167** (2.14)	0.144** (2.39)
CP	0.146** (2.09)	0.075 (0.87)	0.102** (1.99)	0.104** (1.97)	0.076 (0.91)	0.104** (2.06)
ROTA	0.808*** (4.29)	–0.524*** (-3.28)	–0.743*** (-4.85)	0.762*** (3.75)	–0.502*** (-2.94)	–0.731*** (-4.55)
EM	–0.436 (-0.89)	–0.356 (-0.85)	–0.046 (-0.11)	0.014 (0.03)	–0.178 (-0.42)	0.012 (0.03)
TI	0.098*** (11.31)	0.013* (1.80)	0.006 (0.80)	0.078*** (8.03)	0.010 (1.29)	0.004 (0.43)
LS	0.105** (2.47)	0.082** (1.99)	0.025 (0.55)	0.108** (2.45)	0.091** (2.58)	0.055 (1.24)
BM	–0.025 (-0.60)	–0.084** (-2.18)	–0.086** (-2.18)	–0.033 (-0.74)	–0.070* (-1.78)	–0.095** (-2.27)
SH	–0.013 (-0.85)	–0.005 (-0.33)	–0.010 (-0.67)	–0.026* (-1.73)	0.004 (0.26)	–0.002 (-0.15)
Constant	13.130 (1.00)	24.680* (1.73)	0.112 (0.01)	0.837 (0.06)	33.450*** (2.67)	8.219 (0.59)
Year fixed effect	YES	YES	YES	YES	YES	YES
Industry fixed effect	YES	YES	YES	YES	YES	YES
*N*	316	257	221	316	257	221
*R* ^2^	0.576	0.105	0.172	0.486	0.140	0.174

t-statistics are reported in parentheses and are based on robust standard errors clustered at the firm level. *P < 0.10. **P < 0.05. ***P < 0.01.

Table 4 reports the moderating effect of IA, which is significantly negatively correlated in columns (1) and (4). This could be because there are information barriers between different industries, which resulted in serious information asymmetry between the M&A parties and affected the M&A performance of the enterprise in the current period. SA *IA in column (4) significantly positively correlated with the current EVA of the target enterprise (*P* < 0.10) and in columns (2) and (5) significantly positively correlated with the short-term M&A value creation of both parties (*P* < 0.01), indicating that in the case of different industries, the existence of SAs can significantly increase the economic added value of both parties and cause short-term M&A value creation. These statistical results show that the use of SAs in different industries had a more significant M&A value creation effect than those in the same industry. Thus, hypothesis 3 was verified.

**TABLE 4:** Moderating effect of industry attributes.

Variables	Acquirer enterprise			Target enterprise		
	MEVA (1)	ΔMEVA_(–1,0)_ (2)	ΔMEVA_(–1,1)_ (3)	TEVA (4)	ΔTEVA_(–1,0)_ (5)	ΔTEVA_(–1,1)_ (6)
SA	0.052** (2.37)	0.057** (2.15)	0.006 (0.26)	0.050* (1.74)	0.088*** (3.32)	0.021 (0.85)
IA	–0.155*** (-6.43)	0.014 (0.66)	0.022 (0.96)	–0.145*** (-6.06)	0.021 (1.01)	0.014 (0.65)
SA*IA	0.043 (1.53)	0.068*** (3.17)	0.010 (0.47)	0.053* (1.75)	0.085*** (3.67)	0.015 (0.62)
CP	0.109* (1.72)	0.045 (0.60)	0.042 (0.67)	0.082* (1.77)	0.042 (0.59)	0.042 (0.66)
ROTA	0.809*** (4.33)	–0.526*** (-3.28)	–0.735*** (-4.74)	0.752*** (3.70)	–0.502*** (-2.93)	–0.721*** (-4.45)
EM	–0.606 (-1.23)	–0.355 (-0.84)	–0.014 (-0.03)	–0.113 (-0.23)	–0.186 (-0.44)	0.025 (0.05)
TI	0.099*** (11.47)	0.013* (1.75)	0.005 (0.62)	0.079*** (8.07)	0.009 (1.24)	0.002 (0.27)
LS	0.106** (2.52)	0.079* (1.92)	0.023 (0.49)	0.107** (2.46)	0.087** (2.48)	0.052 (1.15)
BM	–0.015 (-0.36)	–0.082** (-2.10)	–0.082** (-2.08)	–0.027 (-0.60)	–0.067* (-1.68)	–0.090** (-2.14)
SH	–0.012 (-0.76)	–0.005 (-0.37)	–0.012 (-0.79)	–0.023 (-1.53)	0.003 (0.19)	–0.004 (-0.28)
Constant	12.930 (1.01)	22.970 (1.64)	–2.162 (-0.14)	0.697 (0.05)	30.500** (2.54)	4.985 (0.36)
Year fixed effect	Yes	Yes	Yes	Yes	Yes	Yes
Industry fixed effect	Yes	Yes	Yes	Yes	Yes	Yes
*N*	316	257	221	316	257	221
*R* ^2^	0.579	0.096	0.145	0.489	0.127	0.142

t-statistics are reported in parentheses and are based on robust standard errors clustered at the firm level. *P < 0.10. **P < 0.05. ***P < 0.01.

## Robustness tests

### Endogeneity test based on propensity score matching method

To overcome the possible endogenous problem of a systematic deviation between the samples with and without SAs, this study utilized the propensity score matching method (PSM) to test the whole sample again. First, taking whether to hire an SA as the explained variable and taking the firm’s market share, audit fees, return on net assets, and total assets as covariates, model (3) was constructed to calculate the propensity score. Second, taking the same year, the same industry, and setting the closest propensity score as the standard, the SA samples were matched 1:1, and 98 samples were obtained. Third, the value creation of M&A parties was utilized as the explained variable, and whether to hire an SA was utilized as the explanatory variable. These variables were utilized to perform another regression. The results are shown in columns (1) and (2) of Table 5. In the statistical results, SA and TEVA were significantly positively correlated (*P* < 0.10), and SA and ΔTEVA_(–1,0)_ were significantly positively correlated (*P* < 0.05). The regression results show that the research conclusion remains unchanged.

**TABLE 5:** Robustness tests.

Variables	TEVA (1)	ΔTEVA_(–1,0)_ (2)	TobinQ (3)	ΔTobinQ_(–1,0)_ (4)	TEVA (5)	ΔTEVA_(–1,0)_ (6)	TEVA (7)	ΔTEVA_(–1,0)_ (8)
SA	0.060* (1.90)	0.073** (2.10)	0.073*** (3.43)	0.092** (2.22)	0.048* (1.69)	0.088*** (3.32)	0.049* (1.72)	0.093*** (3.84)
*R* ^2^	0.536	0.278	0.283	0.043	0.481	0.123	0.482	0.138
*N*	95	85	316	257	316	257	316	257

t-statistics are reported in parentheses and are based on robust standard errors clustered at the firm level. *P < 0.10. **P < 0.05. ***P < 0.01.


(3) $$S\,A=\beta_{0}+\beta_{1}\,M\,s\,h\,a\,r\,e+\beta_{2}\,A\,c\,o\,s\,t+\beta_{3}\,R\,O\,E+\beta_{4}\,T\,a\,s\,s\,e\,t+\varepsilon$$

### Redefinition of mergers and acquisitions value creation

Tobin Q is a common index that is used to measure enterprise value. This study utilized Tobin Q and ΔTobin Q_(–1,0)_ to replace the explained variable, and then another regression analysis was performed on the model. The regression results are shown in columns (3) and (4) of Table 5. The results show that the SAs significantly positively correlated with Tobin Q and ΔTobin Q_(–1,0)_ at least to the level of 5%.

### Redefinition of shared auditors

During the process of SAs, it is possible to change the CPAs, although they still remain in the same accounting firm. Owing to differences in the abilities, preferences, and resources among CPAs, the effect of SAs at transmitting information could be affected, which would result in a deviation of the regression results. Therefore, this study defined the SA as follows: both sides of the M&A parties employed at least one same CPA during the previous year and the current year of the completion of M&A, and the regression analysis was performed again. The regression results in columns (5) and (6) of Table 5 show that SAs with TEVA and ΔTEVA_(–1,0)_ significantly positively correlated.

### Consideration of the impact of differences in corporate governance on enterprise value creation

The separation of two rights is the separation of control and ownership owned by the actual controller of the enterprise, which originates from the fact that the actual controller controls the enterprise in the form of an equity pyramid. The separation of two rights could result in a hollowing out effect and the supporting effect of enterprise value (Lin et al., 2011; Wu, 2019). Therefore, the degree of separation of two rights could affect the value creation of M&A. Therefore, this study further controlled the impact of the separation of two rights on M&A value creation. The regression results in columns (7) and (8) of Table 5 show that the conclusion of this study is still valid after considering the degree of separation between two rights.

## Conclusion

Based on the M&A events completed by Chinese A-share listed companies from 2011 to 2018, this study examined the impact of SAs on the M&A value creation of acquirer and target enterprises. The results show that SAs significantly positively impacted the current economic value-added and short-term M&A value creation of both parties, but they had no significant impact on long-term M&A value creation. The addition of moderating variables of whether it belonged to related M&A and industry attributes showed that compared with related M&A and in the same industry, SAs are more effective at value creation in unrelated M&A and in different industries.

The research findings contribute to the increasing literature on M&A value creation of SAs (Cai et al., 2016; Dhaliwal et al., 2016; Chircop et al., 2018; Bedford et al., 2022). The unique contribution of this study is to explore the value creation effect of SAs from the perspective of a weak form efficient market. It enriches the scope of application of the conclusions on SAs and promotes the research of SAs on the value creation of both parties in M&A. As an important form of weak social relationship network, SAs are the high-quality information sources for both parties of M&A, increase the information flow of both parties, and are conducive to improving the accuracy of resource acquisition and the success rate of M&A. The function of SAs at transmitting information exists not only in countries with perfect capital markets but also in weak form efficient capital markets. Whether the capital market is perfect or not is not the decisive factor for SAs to play the role of information bridge. Another contribution of this study is to enrich the measurement indicators of M&A value creation. This study uses a series of EVA indicators that can directly measure the real value creation of enterprises, while previous studies have used CAR or financial indicators such as ROA and EPS. In fact, EVA not only considers the compensation for the cost of equity capital but also corrects the distortion of accounting rules through accounting adjustment (Chi and Zou, 2015), which can objectively and truly reflect the value creation of enterprises to the maximum extent (Lu, 2012). Using the EVA series of indicators can better evaluate the value creation effect of SAs in a weak form efficient market.

This study also provides useful information for decision-makers of enterprises. M&A is usually a large and important transaction, and it is also one of the most challenging strategic activities implemented by an enterprise (Friedman et al., 2016). Once it fails, it will bring significant losses. Accurately obtaining and identifying the priority and asymmetric information of the other party’s available resources and reducing the uncertainty of M&A are crucial to the matching and integration of resources of both parties. In addition to early stage research, news media, asset appraisal institutions, law firms, and personal social networks of senior management, SAs are also high-quality information sources for enterprise M&A. During the audit process, the auditors can fully understand the business status and sustainable operation ability, and grasp the current business performance, future use of assets, and other private information of the enterprise. The decision-makers of enterprises should attach importance to the information channel of SAs, and obtain important information before, during, and after M&A through direct and indirect communication with SAs.

In addition, the findings of this study can also serve as reference for the policy-making of M&A regulatory authorities. At present, M&A between upstream and downstream enterprises in the same industry is still the main form of M&A of listed companies, but cross-industry and diversified M&A has become an important trend (Fang, 2019). The information asymmetry between the two sides of M&A will hinder the implementation of M&A and reduce the success rate and performance of M&A. To realize the functions of M&A in optimizing resource allocation, promoting industrial upgrading, and structural adjustment, the information exchange mechanism between M&A parties should be optimized. The regulatory authorities of M&A should strengthen the information disclosure responsibilities of both parties and improve the regulatory rules system. At the same time, the regulatory authorities should give full play to the information bridge function of the intermediary institutions. By regulating the practice behavior, improving professional competence and mitigating moral hazards, intermediary institutions can provide high-quality M&A information for both parties.

Auditors need to keep the insider knowledge that they are privy to secret during the execution of the audit business. Thus, it seems to violate the principle of independence for auditors to provide information related to M&A for both parties, but this study did not investigate this. Because the violation of the independence principle could affect the authenticity and accuracy of financial reporting results, the conflict of interest and its impact on SAs in the process of enterprise M&A is an interesting research direction for the future.

## Data availability statement

Publicly available datasets were analyzed in this study. This data can be found here: https://www.gtarsc.com/.

## Author contributions

The author confirms being the sole contributor of this work and has approved it for publication.

## Footnotes

1 Li and Pan (2016) found that Chinese enterprises that once employed accounting firms will still employ accounting firms in subsequent M&A, and the proportion of accounting firms employed in M&A is increasing year by year.

2 M&A transactions are the key audit matters that CPAs focus on. Wu et al. (2019) found that 7,217 key audit matters were disclosed in the annual audit report of China’s A-share Listed Companies in 2017, including 371 key matters related to M&A and reorganization, ranking third in the number.
